# Class II Correction with Microimplant Supported Molar Distalization: A Report of Two Cases

**DOI:** 10.1155/2022/2679318

**Published:** 2022-07-11

**Authors:** Zouhair Skaf, Fidèle Nabbout

**Affiliations:** Department of Orthodontics, School of Dentistry, Lebanese University, Beirut, Lebanon

## Abstract

**Introduction:**

Orthodontic treatment of class II malocclusion with conventional treatment modalities can be challenging for the clinician. The use of microimplants to obtain absolute anchorage has become very popular in recent years especially in noncompliant patients. Microimplants are convenient, save time, and produce good treatment results with no need for patient cooperation. A special approach for class II correction with microimplant supported molar distalization has been developed by the authors and is illustrated through two clinical cases. *Description*. For each clinical case, 0.022” preadjusted brackets were bonded on both arches except on the maxillary first and second premolars with bands on the first and second molars. After leveling and alignment, a 0.017^”^ × 0.025^”^ stainless steel wire was fitted on the upper arch, and two microimplants were placed bilaterally between the maxillary second premolar and the first molar. Open coil springs were inserted in the upper archwire on both sides and compressed via a steel ligature on sliding hooks to the microimplants pushing distally simultaneously the first and second maxillary molars. En-masse retraction of the maxillary anterior teeth was then carried out on a 0.019^”^ × 0.025^”^ stainless steel closing loop archwire while the posterior segment was anchored to the microimplant with a steel ligature to the first premolar.

**Results:**

Class I canine and molar relationship were achieved, and an ideal occlusion was established. Both ANB and FMA angles decreased by 1° due to the counterclockwise rotation effect of the maxillomandibular complex. Skeletal and dental results remained stable three years later.

**Conclusion:**

Maxillary molar distalization using coils and buccal microimplants can be regarded as an effective technique in a relatively short time and might be considered a breakthrough in the treatment of class II malocclusions. Microimplants enable the clinician to perform a nonextraction treatment in noncompliant patients who would alternatively be treated only with extractions.

## 1. Introduction

Over the past decade, the use of microimplants is becoming a standard practice in orthodontics. Their potential has been demonstrated repeatedly during orthodontic treatment for different types of movement: molar distalization [1–4], molar protraction [5, 6], incisor or molar intrusion [7, 8], incisor or molar extrusion [9, 10], en-masse retraction of anterior teeth [11, 12], and maintaining anchorage in extraction cases [13–15]. Microimplant-based distalization of maxillary molars can help facilitate correction of class II malocclusions by avoiding premolar extraction, decreasing the need for surgery in specific cases, and reducing patient compliance while keeping usual goals of treatment [ 16, 17].

**Figure 1:**
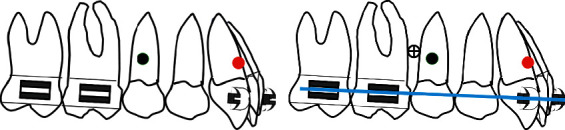
(a) Typical strap up (0.022^”^ × 0.028^”^ slot), maxillary arch is bonded bypassing the premolars; black circle is the center of resistance of the whole arch; red circle is the center of resistance of the anterior segment. (b) After leveling and alignment, a 0.017^”^ × 0.025^”^ stainless steel archwire is inserted and a microimplant (cross circle) is placed bilaterally between the second premolar and the first molar.

**Figure 2:**
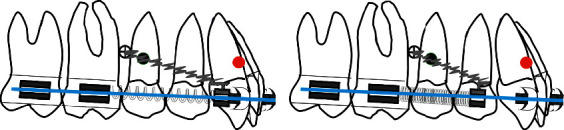
(a) An open coil is activated via a steel ligature from the microimplant to a sliding hook. Care should be taken to select the length of the coil equal to the distance distal canine bracket-mesial first molar tube so that in case of failure of the microimplant no anterior anchorage loss will occur. (b) Open coil is compressed against the molars pushing them distally.

**Figure 3:**
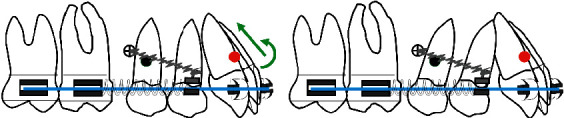
(a) When the open coil is extended, simultaneous distalization of the first and second molar will result and since it is a rigid system with indirect mechanics represented by the steel ligature, the whole system will rotate around a fixed point represented by the microimplant introducing maxillary incisor intrusion and counterclockwise rotation of the maxillary arch (green arrows). (b) Spontaneous distal drifting of premolars will follow.

**Figure 4:**
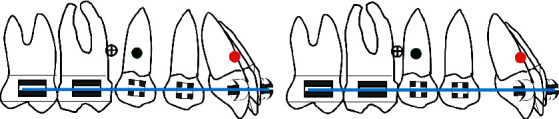
(a) At that stage the microimplant is displaced distally according to its initial angulation, if it is parallel to the long axis of the tooth there is no need to displace it, if it is perpendicular to the bone surface, it is recommended to relocate it distally to avoid any contact with the premolar roots; then, the premolars are bonded and leveled. (b) Uprighting of premolars and space closure posteriorly.

**Figure 5:**
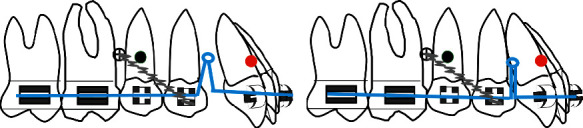
(a) En-masse retraction of the maxillary anterior teeth with loop mechanics while the posterior segment is anchored to the microimplant with a steel ligature to the first premolar. (b) End of en-masse retraction.

The authors have developed a special procedure for maxillary molar distalization illustrated in the following drawings (Figures 1–5).

## 2. Case 1

### 2.1. Diagnosis and Etiology

**Figure 6:**
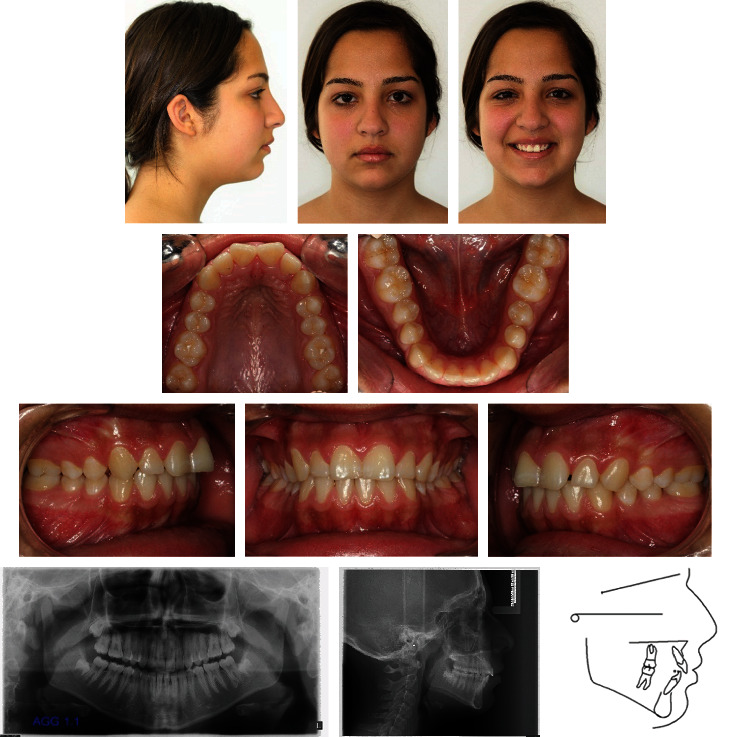
Case 1. 15-year-old female patient with a class II molar relationship subdivision right, shifted maxillary midline, and slightly proclined upper and lower incisors before treatment.

A 15-year-old female presented with the chief complaints of maxillary incisor crowding and painful right jaw joint during chewing and opening but not during lateral excursion (Figure 6). Clinical evaluation found a convex profile and a normal nasolabial angle. The maxillary midline was shifted 2 mm to the left compared with the facial midline due probably to a premature loss of the temporary left canine. She exhibited a class II molar relationship subdivision right, a class II canine on the same side, and an overbite of 2 mm. The maxillary first molars were mesially rotated with no crowding on the mandibular arch. The patient had TMJ pain and clicking on the right side. The cause of this TMJ disorder was not clear and may be due to a combination of factors. She was wearing a splint for several months with no signs of improvement. Cephalometric analysis (Table 1) indicated a skeletal class II malocclusion (ANB = 5°, Wits appraisal = +2 mm) with a retrognathic mandible (SNB = 72°) and slightly proclined upper and lower incisors (U1 − FH = 114°, IMPA = 95°). The vertical facial pattern was within normal range (FMA = 28°). The panoramic radiograph confirmed the presence of all teeth, including the third molars still unerupted and at the crown completion stage.

### 2.2. Treatment Objectives

Treatment objectives were to derotate the maxillary first molars, establish a class I canine and molar relationship on the right side, correct the maxillary midline, and relieve the TMJ pain. Distal movement of the maxillary dentition on the right side was planned by using microimplant anchorage.

### 2.3. Treatment Progress

**Figure 7:**
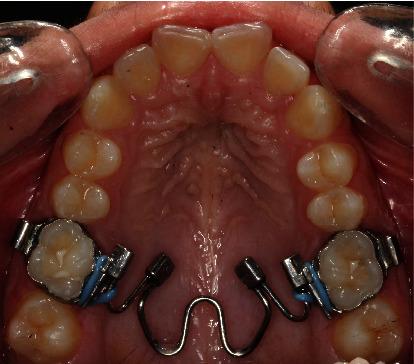
Case 1. Derotation of maxillary first molars with a nickel-titanium molar rotator.

Derotation of maxillary first molars was initiated with a nickel-titanium molar rotator (Registered trademark of Ortho Organizers Inc., Carlsbad, CA; http://www.orthoorganizers.com.) (Figure 7).

**Figure 8:**
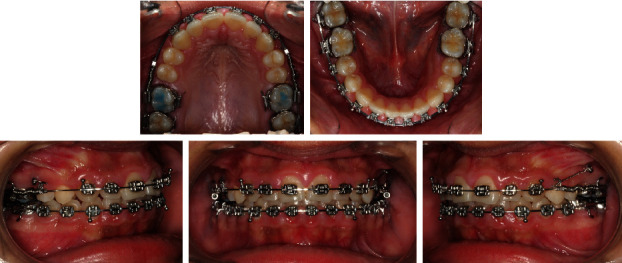
Case 1. Simultaneous distalization of maxillary first and second molars, a space mesial to the maxillary right first molar was noticed.

The molar rotator is a thermally activated nickel-titanium appliance providing predictable molar derotation and expansion. The correction of maxillary first molars rotation is recommended as a first procedure, prior to their distalization [18]. Three months later, the molar rotator was removed and Roth-prescription 0.022” brackets (Mini-Taurus, registered trademark of Rocky Mountain Orthodontics, Denver, CO; http://www.rmortho.com.) were bonded on both arches except on the maxillary first and second premolars with bands on the first and second molars. Leveling and alignment were carried out with sequential archwires, progressing up to full-size 0.020^”^ × 0.025^”^ stainless steel on the lower arch and 0.017^”^ × 0.025^”^ stainless steel on the upper arch. After five months of treatment, the patient was asked to extract her upper third molars, and 1.4 mm × 8 mm microimplants (AbsoAnchor, registered trademark of Dentos, Inc., Daegu, Korea; http://www.dentos.co.kr.) were placed bilaterally between the second premolar and the first molar. The purpose of placing a microimplant on the left side was to avoid any canting of the occlusal plane. Open coil springs were inserted in the upper archwire on both sides and compressed via a steel ligature on sliding hooks to the microimplants pushing distally simultaneously the first and second maxillary molars. The activation of the springs was greater on the right side to correct the class II molar relationship. Four months later, a space mesial to the maxillary right first molar was noticed confirming the distalization of the maxillary first and second molars (Figure 8). The maxillary first and second premolars drifted distally spontaneously and were bonded at a later stage. En-masse retraction of the maxillary anterior teeth was then carried out on 0.019^”^ × 0.025^”^ stainless steel closing loop archwire while the posterior segment was anchored to the microimplant with a steel ligature to the first premolar with no use of class II elastics. After 18 months of treatment, all appliances were removed, upper and lower fixed lingual retainers were bonded, and wraparound retainers were also delivered. The patient was asked to extract her lower third molars.

### 2.4. Treatment Results

**Figure 9:**
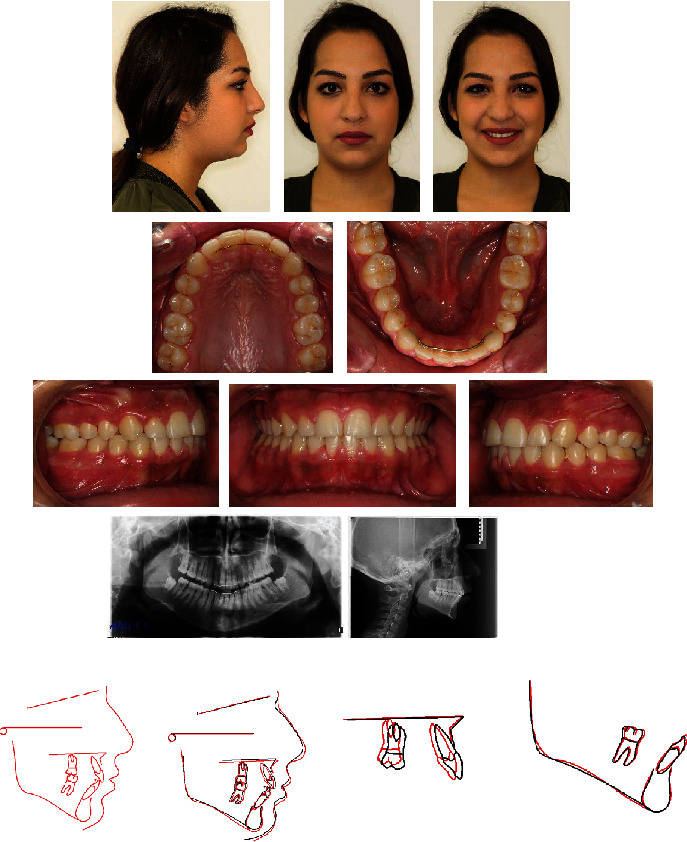
Case 1. (a) Patient after 18 months of treatment. (b) Superimposition of pretreatment (black) and posttreatment (red) cephalometric tracings.

**Figure 10:**
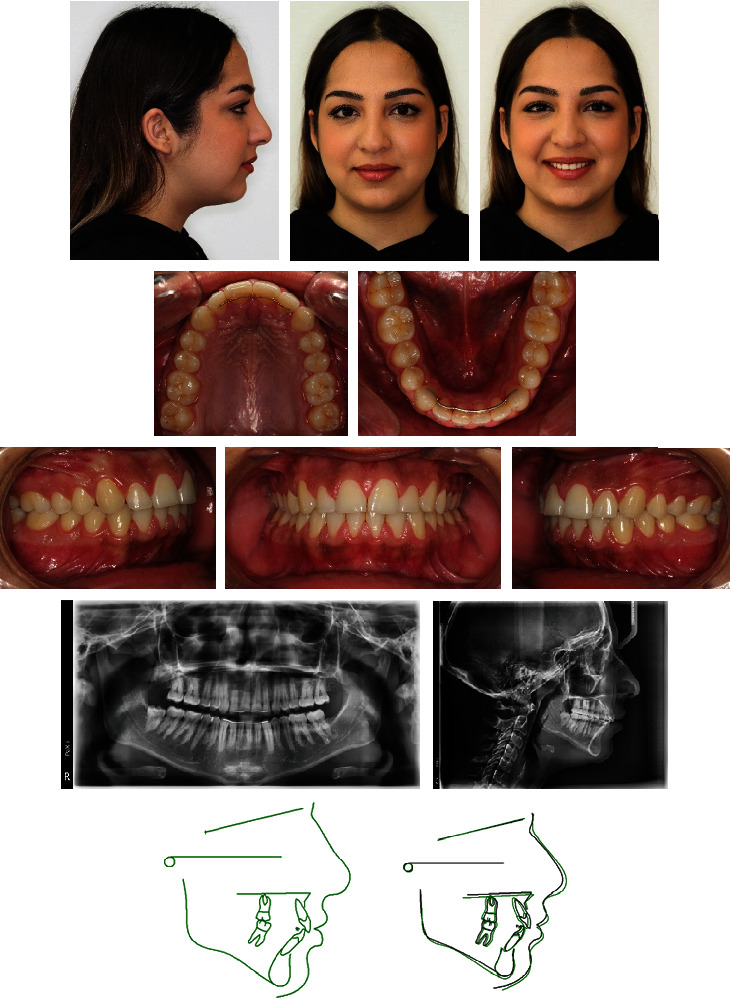
Case 1. Patient three years after treatment.

Posttreatment records showed an improved profile and occlusion (Figure 9). Class I canine and molar relationship were achieved on the right side, and an ideal occlusion with coincident midlines was established. There was also a complete relief of TMJ symptoms. Root parallelism was good, and both ANB and FMA angles decreased by 1° due to the counterclockwise rotation effect of the maxillomandibular complex (Table 1). Skeletal and dental results remained stable three years later (Figure 10).

## 3. Case 2

### 3.1. Diagnosis and Etiology

**Figure 11:**
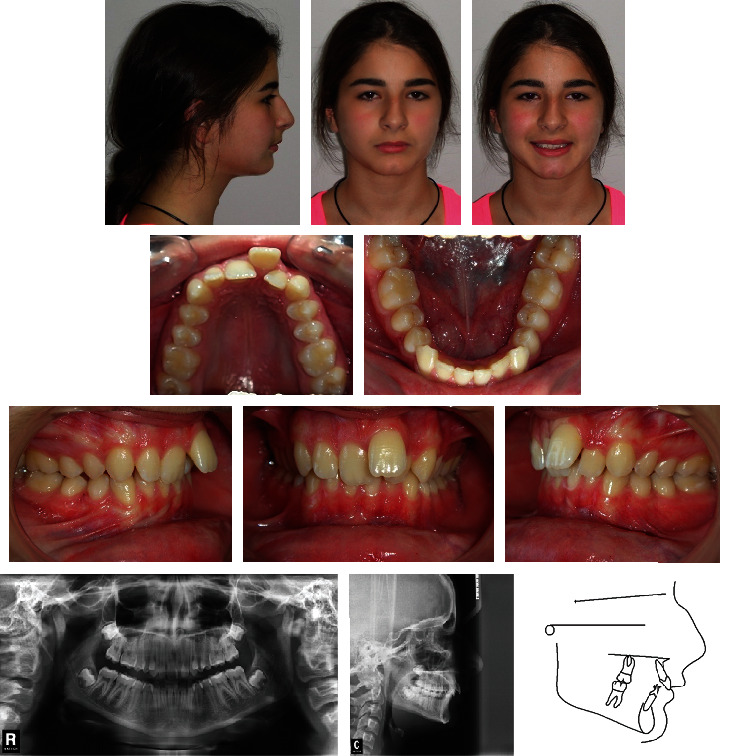
Case 2. 15-year-old female patient with bilateral class II canine and molar relationships, shifted maxillary midline, and blocked-out buccally upper left central incisor before treatment.

A 15-year-old female presented with the chief complaint of severe maxillary incisor crowding (Figure 11). Clinical evaluation found a concave profile and an obtuse nasolabial angle. The maxillary midline was shifted 3 mm to the left compared with the facial midline, and the upper left central incisor was blocked-out buccally. The patient had a class II canine and molar relationships on both sides, with an overbite of 6 mm. There was a localized overjet of 4 mm at the level of the upper left central incisor. The mandibular incisors were slightly crowded. Cephalometric analysis (Table 2) indicated a skeletal class II malocclusion (ANB = 5°, Wits appraisal = +2.5 mm) with a retrognathic mandible (SNB = 76°), and slightly proclined upper and lower incisors (U1 − FH = 112°, IMPA = 96°). The growth pattern was horizontal (FMA = 21°). The panoramic radiograph confirmed the presence of all teeth, including the third molars still unerupted and at the crown completion stage.

### 3.2. Treatment Objectives

Treatment objectives were to resolve the crowding and align the upper left central incisor, produce a class I canine and molar relationships, correct the maxillary midline, reduce the overbite, and preserve facial esthetics. A nonextraction treatment option was decided in order not to alter the soft-tissue profile. Distal movement of the maxillary dentition was planned by using microimplant anchorage.

### 3.3. Treatment Progress

**Figure 12:**
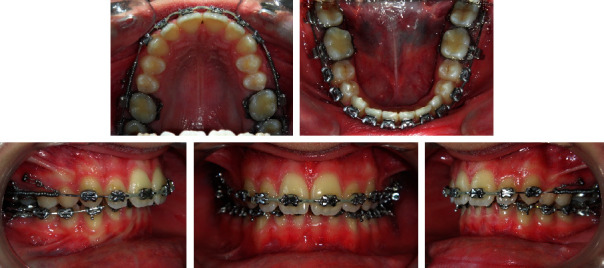
Case 2. Simultaneous distalization of maxillary first and second molars.

MBT-prescription 0.022” brackets (Mini Uni-Twin, registered trademark of 3 M, Monrovia, CA; http://www.3M.com.) were bonded on the upper arch except on the first and second premolars bypassing the left central incisor with bands on the first and second molars. Leveling and alignment were carried out with sequential archwires, progressing up to 0.017^”^ × 0.025^”^ stainless steel. After six months of treatment, 1.4 mm × 8 mm microimplants (AbsoAnchor, registered trademark of Dentos, Inc., Daegu, Korea; http://www.dentos.co.kr.) were placed bilaterally between the second premolar and the first molar. Open coil springs were inserted in the upper archwire on both sides and compressed via a steel ligature on sliding hooks to the microimplants pushing distally simultaneously the first and second maxillary molars. Four months later, after enough space had been created for the upper left central incisor, it was bonded and followed by the bonding of the mandibular arch (Figure 12). The maxillary first and second premolars drifted distally spontaneously and were bonded at a later stage. En-masse retraction of the maxillary anterior teeth was then carried out on 0.019^”^ × 0.025^”^ stainless steel closing loop archwire while the posterior segment was anchored to the microimplant with a steel ligature to the first premolar with no use of class II elastics. After 24 months of treatment, all appliances were removed, upper and lower fixed lingual retainers were bonded, and wraparound retainers were also delivered. The patient was asked to extract her four third molars.

### 3.4. Treatment Results

**Figure 13:**
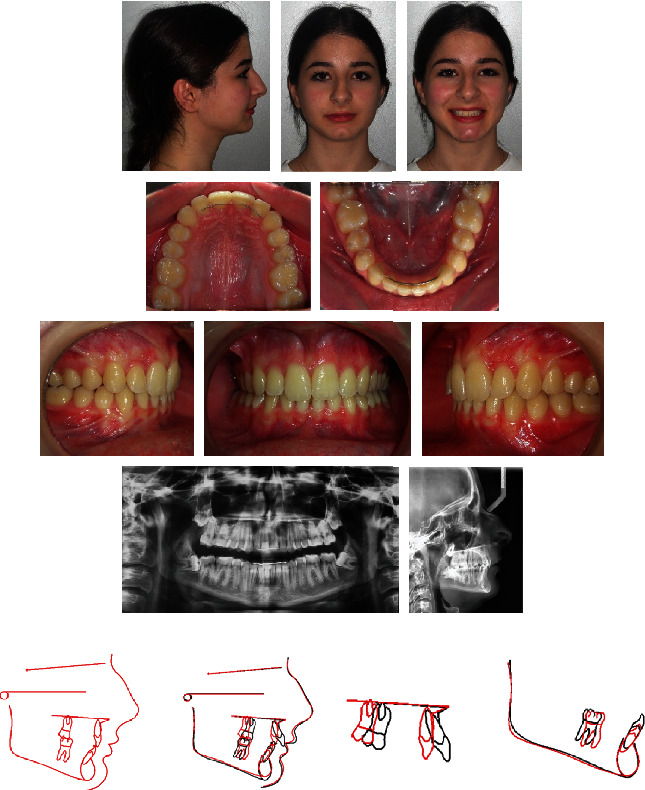
Case 2. (a) Patient after 24 months of treatment. (b) Superimposition of pretreatment (black) and posttreatment (red) cephalometric tracings.

**Figure 14:**
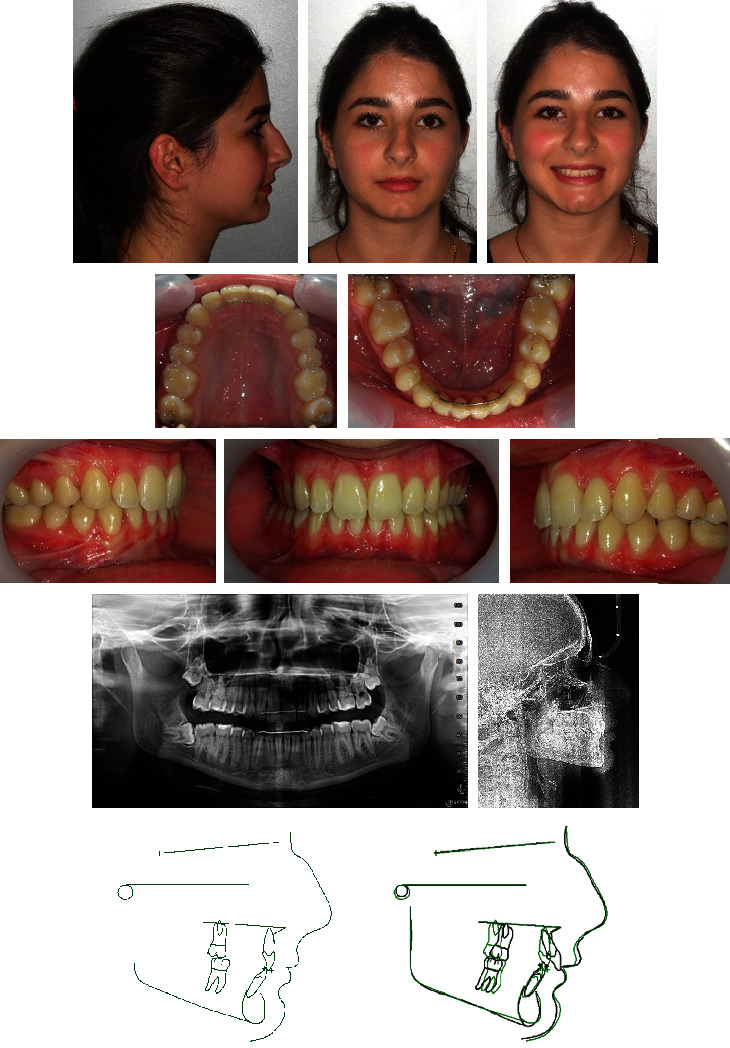
Case 2. Patient three years after treatment.

Posttreatment records showed an improved profile and occlusion (Figure 13). Bilateral class I canine and molar relationships were achieved, and an ideal occlusion with matching midlines was established. The upper left central incisor showed no signs of root resorption. Root parallelism was acceptable, and both ANB and FMA angles decreased by 1° due to the counterclockwise rotation effect of the maxillomandibular complex (Table 2). Skeletal and dental results remained stable three years later (Figure 14).

## 4. Discussion

In the two cases presented here, both maxillary first and second molars are distalized simultaneously in a bodily movement. Although the distalizing force does not pass through the center of resistance of the molars, by moving teeth together, individual tooth movements, rotations, and tipping are prevented [19]. For the molar distalization stage and based on our clinical experience, using a 0.017^”^ × 0.025^”^ stainless steel archwire in a 0.022” slot could be considered an acceptable combination of wire stiffness and wire sliding. In both cases, indirect anchorage was used with the steel ligature performing as a rigid link, and the whole system rotated around a fixed point represented by the microimplant introducing maxillary incisor intrusion and counterclockwise rotation of the maxillary arch [17] (Figure 3(a)). Therefore, this type of mechanics is mainly indicated in class II deep bite cases. The use of class II elastics should be avoided to prevent an increase in vertical facial dimension and proclination of the lower incisors [20]. Class II elastics were not used in our cases, instead class III elastics against microimplants might be used if controlling the lower incisor position is needed [21].

When the anatomic situation is favorable and allows for a vertical placement of the microimplants, relocating them distally to provide an unrestricted movement of the premolars is not necessary [22].

For case 1, derotation of maxillary first molars prior to their distalization helped partially in the class II molar correction, since more than 85% of class II malocclusions involve some mesiopalatal rotation of the first molar crowns [ 23, 24], a situation that exaggerates the class II relationship by locking the mandible in a retrusive position [25].

When distalizing maxillary first molars, there is no difference in the amount of distalization in patients with erupted and unerupted maxillary second molars [26].

For case 2, upper third molars were not extracted at the start of treatment since their buds were very high and cannot impede molar distalization.

## 5. Conclusions

Maxillary molar distalization using coils and buccal microimplants can be regarded as an effective technique in a relatively short time [27].

Despite the currently limited evidence supporting the influence of factors predictive of sagittal stability following class II malocclusion treatment, the two cases presented here provides clinical evidence of stability three years after treatment [28].

Maxillary molar distalization using microimplants might be considered a breakthrough in the treatment of class II malocclusions. They enable the clinician to perform a nonextraction treatment in noncompliant patients who would alternatively be treated only with extractions. The orthodontist must select the most appropriate distalization device on an individual base after a careful diagnosis and treatment plan.

## Figures and Tables

**Table 1:** Case 1 cephalometric analysis.

	Norm	Pretreatment	Posttreatment
SNA	82°	77°	77°
SNB	80°	72°	73°
ANB	2°	5°	4°
Wits appraisal	0 mm	+2 mm	+1.5 mm
FMA	25°	28°	27°
IMPA	88°	95°	98°
FMIA	67°	57°	55°
U1-FH	107°	114°	112°
U1-L1	135°	124°	123°
Occlusal plane	10°	10°	10°
*Z* angle	75°	68°	67°
Upper lip	mm	12 mm	12 mm
Total chin	mm	12 mm	12 mm

**Table 2:** Case 2 cephalometric analysis.

	Norm	Pretreatment	Posttreatment
SNA	82°	81°	80°
SNB	80°	76°	76°
ANB	2°	5°	4°
Wits appraisal	0 mm	+2.5 mm	+1.5 mm
FMA	25°	21°	20°
IMPA	88°	96°	98°
FMIA	67°	63°	62°
U1-FH	107°	112°	93°
U1-L1	135°	128°	148°
Occlusal plane	10°	12°	10°
Z angle	75°	66°	70°
Upper lip	mm	11 mm	15 mm
Total chin	mm	13 mm	14 mm
